# The Role of the Liquid Biopsy in Decision-Making for Patients with Non-Small Cell Lung Cancer

**DOI:** 10.3390/jcm9113674

**Published:** 2020-11-16

**Authors:** D. Akhoundova, J. Mosquera Martinez, L. E. Musmann, C. Britschgi, C. Rütsche, M. Rechsteiner, E. Nadal, M. R. Garcia Campelo, A. Curioni-Fontecedro

**Affiliations:** 1 Department of Medical Oncology and Hematology, Comprehensive Cancer Center Zurich, University Hospital Zurich and University of Zurich, 8091 Zurich, Switzerland; dilara.akhoundovasanoyan@usz.ch (D.A.); Christian.Britschgi@usz.ch (C.B.); Cyrill.Ruetsche@usz.ch (C.R.); 2 Department of Medical Oncology, University Hospital A Coruña, 15006 A Coruña, Spain; Joaquin.Mosquera.Martinez@sergas.es (J.M.M.); ma.rosario.garcia.campelo@sergas.es (M.R.G.C.); 3 Department of Internal Medicine, University Hospital Zurich, 8091 Zurich, Switzerland; LydiaEliane.Musmann@usz.ch; 4 Department of Pathology and Molecular Pathology, University Hospital Zurich, 8091 Zurich, Switzerland; Markus.Rechsteiner@usz.ch; 5 Department of Medical Oncology, Catalan Institute of Oncology, 08908 L’Hospitalet de Llobregat, Barcelona, Spain; esnadal@iconcologia.es

**Keywords:** liquid biopsy, lung cancer, resistance mechanisms, targeted therapy

## Abstract

Liquid biopsy is a rapidly emerging tool of precision oncology enabling minimally invasive molecular diagnostics and longitudinal monitoring of treatment response. For the clinical management of advanced stage lung cancer patients, detection and quantification of circulating tumor DNA (ctDNA) is now widely adopted into clinical practice. Still, interpretation of results and validation of ctDNA-based treatment decisions remain challenging. We report here our experience implementing liquid biopsies into the clinical management of lung cancer. We discuss advantages and limitations of distinct ctDNA assay techniques and highlight our approach to the analysis of recurrent molecular alterations found in lung cancer. Moreover, we report three exemplary clinical cases illustrating the complexity of interpreting liquid biopsy results in clinical practice. These cases underscore the potential and current limitations of liquid biopsy, focusing on the difficulty of interpreting discordant findings. In our view, despite all current limitations, the analysis of ctDNA in lung cancer patients is an essential and highly versatile complementary diagnostic tool for the clinical management of lung cancer patients in the era of precision oncology.

## 1. Introduction: State of the Art and Uses of Liquid Biopsy in Lung Cancer

Liquid biopsy is an increasingly relevant diagnostic tool in the management of patients with lung cancer [1]. This method is based on detection of circulating cancer cell markers in liquid samples, predominantly plasma. Such markers include circulating free DNA (cfDNA), circulating tumor DNA (ctDNA), exosomes, circulating tumor cells (CTCs), microRNAs, as well as antigens and antibodies derived from cancer cells [2]. Nowadays, liquid biopsy is mainly used in lung cancer for patients with metastatic disease in order to monitor response or to detect emerging resistance mechanisms, while further uses such as early tumor detection are still under investigation [3,4]. Over the last years, an increasing number of clinical trials in lung cancer have incorporated the use of liquid biopsy, in addition to tissue genomic testing. However, still limited data are available correlating results from liquid biopsies with response to molecularly targeted treatment or resistance. Different assays can be used, such as real-time polymerase chain reaction (PCR), digital droplet PCR (ddPCR), and next-generation sequencing (NGS) [5,6]. For the most frequently targeted genes in lung cancer, such as *EGFR*, a high concordance rate of more than 90% has been shown between alterations detected in liquid biopsy and matched tissue samples [7,8]. However, less frequent genomic alterations lack such standardization. Additionally, the analytical sensitivity is variable, depending on the implemented assay [9]. Our work reports a series of controversial clinical cases and reviews related literature data.

## 2. Available Assays and Target Genomic Alterations in Lung Cancer

### 2.1. Liquid Biopsy Assays

For genomic profiling of lung cancer, several assays, which can be used in clinical routine and for research purposes, are available to date. This leads to a broad spectrum of alternatives (Table 1) to evaluate the most common genes of interest: *EGFR*, *ALK*, *BRAF*, *KRAS*, *NRAS*, *MET*, *ERBB2*, *ROS1,* and *NTRK* (Table 2).

#### 2.1.1. Real-Time PCR

During real-time PCR, also designed as quantitative PCR (qPCR), specific DNA or complementary DNA (cDNA) sequences undergo amplification and thermal cycling, with measurement of obtained DNA quantity after completion of each cycle. Main advantages of qPCR are the speed and cost effectiveness, as compared to other techniques. Single nucleotide variants (SNVs), deletions, fusions, or markers of DNA methylation can be detected [10]. As disadvantages, only limited DNA sequences can be analyzed in the same reaction. Additionally, the limits of detection and limits of quantification vary depending on the calibration of the assay used [11]. If Therascreen or the cobas^®^ kits are used, usually an allele frequency of at least 1% is required due to high rate of false negative results [10]. The cobas^®^ EGFR Mutation Test v2, approved by FDA in 2016, is a real-time PCR assay that interrogates 42 mutations within *EGFR* exons 18, 19, 20, and 21, including the resistance mutation p. T790M, as well as the less common exon 20 p. S768I and exon 21 p. L861Q mutations [12]. In the phase III ENSURE trial [13], which evaluated the efficacy and safety of first-line erlotinib as compared to chemotherapy with cisplatin and gemcitabine in patients with advanced non-small cell lung cancer (NSCLC) harboring an *EGFR* activating mutation, plasma samples were collected from 517 patients. In parallel to tissue biopsy analysis, cobas^®^ EGFR Mutation Test v2 was used to assess the presence of *EGFR* activating mutations in plasma. In patients without mutations of *EGFR* (Del19 and p. L858R), the liquid biopsy resulted negative in 98.2% of cases (95% confidence interval (CI): 95.4–99.3%) as compared with tissue testing. In patients with tumors harboring an *EGFR* mutation, however, sensitivity of the plasma tests was only 76.7% (95% CI: 70.5–81.9%). Based on this trial, the cobas^®^ EGFR Mutation Test v2 received FDA approval for plasma testing of EGFR Del19 and p. L858R mutations in order to identify patients eligible for treatment with erlotinib. However, it was specified in the approval that patients with negative liquid biopsy results should undergo tissue testing. Additionally, in the ENSURE trial, detection of cfDNA for EGFR Del19 and p. L858R mutations positively correlated with progression-free survival (PFS) [13].

In the pooled population of the AURA2 study, a phase 2 trial that evaluated the safety and efficacy of osimertinib in the second-line setting for patients harboring the p. T790M resistance mutation [14], liquid biopsy sampling with analysis of cfDNA was performed. cfDNA was analyzed with cobas^®^ EGFR Mutation Test v2 and compared with available tissue biopsies. For the EGFR p. T790M mutation, the liquid biopsy test had a sensitivity of 61% (95% CI: 57–66%), a specificity of 79% (95% CI: 70–85%), and a concordance of 65% (95% CI: 61–69%), as compared to the tissue assay. For the EGFR Del19 and p. L858R mutations, the sensitivity was of 85% and 76%, the specificity of 98% for both mutations, and the concordance with tissue biopsy was 90% and 91%, respectively [15].

As part of a comparative study evaluating the quality of assessment of *EGFR* mutations in plasma, across seven Korean laboratories, the level of detection (LOD) of the cobas^®^ EGFR Mutation Test v2 was assessed. LOD was of 5–27 copies/mL for the EGFR Del19 mutation, 35–70 copies/mL for the pL858R mutation, and 18–36 copies/mL for the resistance p. T790M mutation [9].

Other assays based on real-time PCR are Idylla^TM^ ctEGFR Mutation Assay, covering 49 mutations in the same four exons of *EGFR* (18–21), as well as Therascreen EGFR RGQ PCR Kit v2, testing 29 mutations. The Therascreen EGFR RGQ PCR Kit v2 has been implemented in the LUX-Lung 3 and 6 trials for assessment of a total of 287 serum and 334 plasma samples from patients harboring an *EGFR* mutation. In the LUX-Lung 3 study, the Therascreen detected *EGFR* mutations in 28.6% of the cases. This detection rate was higher (60.5%) in the LUX-Lung 6 trial [16].

#### 2.1.2. Digital Droplet PCR

DdPCR improves quantification of amplified DNA sequences by dispersing the sample in uniform droplets [17,18]. The major advantage of this technique is the increased sensitivity, which enables the detection of as low allele fractions as 0.005 –0.1% [19,20]. A comparative study evaluated the performance of ddPCR, real-time PCR, and MALDI-TOF mass spectrometry in detecting EGFR p. T790M mutations in tissue biopsies. Additionally, the three methods were compared with results of ultradeep NGS using SiRe^®^. ddPCR and NGS could more frequently identify positive cases with very low mutant-allele frequencies (ranging from 0.07% to 0.38%) [21]. Similarly to real-time PCR, a disadvantage of ddPCR is the limited number of mutant DNA sequences that can be analyzed within one assay.

#### 2.1.3. Next-Generation Sequencing

NGS allows to screen simultaneously multiple genes, requiring, however, the presence of a higher allele frequency, as compared to ddPCR [22,23]. Oncomine^TM^ Lung cfDNA Assay is an NGS-based liquid biopsy assay that interrogates the most frequent mutations in NSCLC across eleven genes. With a LOD between 0.1% and 1% of allele frequency, the assay showed a sensitivity of 87.5% and a specificity of 100% for hotspot mutations. For an allele frequency of 0.1%, sensitivity of the assay was of 91.7% for point mutations, being lower (75%) for detection of indels. For the same allele frequency, specificity was 100% for both point mutations and indels [23].

GUARDANT360^®^ Circulating Tumor DNA Assay is another NGS-based test with coverage of 54–73 genes, depending on the version of the test. For SNVs, the GUARDANT360^®^ 73 gene-panel has a sensitivity of 100% with a positive predictive value (PPV) of 99.2% for an allele frequency of ≥0.25%, and a sensitivity of 63.8% with a PPV of 96.3% for an allele frequency of 0.05–0.25%. As compared to tissue NGS analysis, plasma assessment with GUARDANT360^®^, pooled with results from FoundationOne^®^ Liquid CDx assay, showed a concordance between 91% and 94.2%, however, in a cohort of 45 patients with breast cancer [24].

### 2.2. Target Genomic Alterations

#### 2.2.1. EGFR Del19 and p. L858R Mutations

The FLAURA phase 3 study compared first-line osimertinib to gefitinib or erlotinib in patients with locally advanced or metastatic NSCLC harboring an activating *EGFR* mutation. In FLAURA, the plasma cobas^®^ EGFR Mutation Test v2 was retrospectively used to assess the presence of the EGFR Del19 and p. L858R mutation in plasma and compared to the tissue testing results. From a cohort of 211 patients with centrally confirmed positive tissue *EGFR* mutational status, 79% had a concordant positive liquid biopsy results with plasma cobas^®^ EGFR Mutation Test. Interestingly, PFS was longer in both treatment arms (osimertinib and gefitinib/erlotinib) in patients with negative cfDNA for *EGFR* mutations (23.5 and 15.0 months, respectively). Moreover, patients with positive cfDNA analysis showed a shorter PFS (15.2 and 9.7 months, respectively) [25]. These data underline the predictive value of cfDNA for EGFR-targeted treatments.

#### 2.2.2. EGFR p. T790M Mutation

The p. T790M mutation of the *EGFR* gene is the main resistance mechanism to first and second generation EGFR-TKIs [26,27]. The liquid biopsy with the cobas^®^ EGFR Mutation Test v2 performed in 419 patients from the AURA3 trial showed that the concordance with tissue biopsies was lower for the EGFR p. T790M mutation than for the other *EGFR* activating mutations (51% for p. T790M, 82% for Del19, and 68% for p. L858R) [28] This underscored the relevance of performing tissue rebiopsies in case of negative plasma results at tumor progression, where a p. T790M is suspected. A meta-analysis reviewing eleven studies using ddPCR for detection of the EGFR p. T790M mutation in cfDNA reported a global sensitivity and specificity in the pooled analysis of 70.1% (95% CI: 62.7–76.7%) and 86.9% (95% CI: 80.6–91.7%), respectively (1). In a comparative evaluation of different assays used for detection of the EGFR p. T790M mutation in plasma samples from the AURA trial, the authors compared in 38 samples the cobas^®^ EGFR Mutation Test, the Therascreen™ EGFR amplification refractory mutation system assay, and two digital assays: Droplet Digital™ PCR and BEAMing digital PCR. The two digital platforms showed a better performance. In the subsequent direct comparison between cobas^®^ EGFR Mutation Test and BEAMing digital PCR in 72 additional plasma samples, the sensitivity of the cobas^®^ EGFR Mutation Test for detection of the p. T790M mutation was of 73%, as compared to the 81% obtained by BEAMing digital PCR. The specificity was of 67% and 58%, respectively, and the concordance between the 2 platforms of 90% [29].

In the liquid biopsy assessment of the p. T790M mutation performed as part of the TIGER-X and TIGER-2 trials, which evaluated the safety and efficacy of rociletinib, a third-generation EGFR TKI, in patients with EGFR mutant lung cancers progressing after first-line TKI, the GUARDANT360^®^ 70-gene NGS panel was used to analyze cfDNA. While primary *EGFR* activating mutations were identified in 93% of the cases, the resistance mutation p. T790M could be detected only in 85% of positive matching tissue samples (Therascreen EGFR RGQ PCR Kit, Qiagen) [30].

In a systematic meta-analysis evaluating the accuracy of ctDNA-based assays to investigate the detection of the p. T790M mutation, a total of 21 studies up to 2018 were reviewed, including 1639 patients. Digital PCR was the most frequently implemented technique, used in 12 out of 21 studies, followed by real-time PCR in 6 studies. NGS was performed in 3 out of 21 studies. The pooled analysis showed a global sensitivity for ctDNA analysis of 0.67 (95% CI: 0.64–0.70), with a pooled specificity of 0.80 (95% CI: 0.77–0.83). The global PPV was 0.85 (95% CI: 0.82–0.87), and the negative predictive value (NPV) was 0.60 (95% CI: 0.56–0.63) [31].

#### 2.2.3. EGFR p.C797S Mutation

The emergence of the resistance mutation p.C797S in exon 20 of the *EGFR* gene constitutes one of the major acquired resistance mechanisms to osimertinib [32]. This mutation could be detected using NGS, followed by targeted ddPCR assays, in cfDNA of NSCLC patients treated within the AURA phase I trial. When performing ddPCR cfDNA analysis in resistant tumors, distinct mutational scenarios have been detected: occurrence of the p.C797S mutation, persistence of the p. T790M mutation without addition of p.C797S, and loss of p. T790M with persistence of the initial *EGFR* mutation [33]. Using cfDNA NGS, the concomitant detection of the original *EGFR* activating mutation together with p. T790M and p.C797S has been reported at progression under treatment with third-generation TKI [34]. Interestingly, the type of resistance mutation has shown to be specific for the inhibitor used: the p. L798I mutation has been reported under treatment with rociletinib, and, while approximately a third of patients under osimertinib showed the p. C797S resistance mutation, this was only detected in 2% of patients treated with rociletinib [35]. Additionally, some RT-PCR-based assays, such as EntroGen ctEGFR Mutation Detection Kit, also enable the detection of this mutation. Although no effective targeted treatment for this resistance mutation is yet available, its detection is gaining relevance since third-generation EGFR inhibitors have become standard for up-front treatment of *EGFR*-mutated NSCLC [36].

#### 2.2.4. ALK

Different liquid biopsy methods can be used to assess the presence of ALK rearrangements in lung cancer patients. Real-time PCR was used in a cohort of 77 patients with NSCLC to detect *ALK* rearrangements. In plasma, the specificity of the assay was 100%, with a sensitivity of 21%. In platelets, the sensitivity increased to 65%, maintaining the 100% of specificity [37]. The lower sensitivity observed for the plasma test could be related to the lower stability of circulating RNA in plasma as compared to RNA extracted from platelets [31].

In a study using real-time PCR to assess *ALK* status in lung cancer, 33 *ALK*-positive and 28 *ALK*-negative patients, assessed by fluorescent in situ hybridization (FISH) break-apart, were enrolled. Plasma, platelets, and tumor tissue were used for RNA extraction and cDNA synthesis. Results of liquid biopsy showed a higher sensitivity (78.8%), specificity (89.3%), and accuracy (83.6%) for detection of *ALK* rearrangements, as compared to the formalin-fixed paraffin-embedded (FFPE) tissue analysis, with 54.5% sensitivity, 78.6% specificity, and 75.5% accuracy. Liquid biopsy was more frequently positive in patients with later sampling, defined as 6 months or later following diagnosis of lung cancer. In the subgroup of 26 patients treated with the ALK-TKI inhibitor crizotinib, patients with platelet-positivity for *ALK* rearrangements had a longer treatment duration (7.2 versus 1.5 months), median PFS (5.7 versus 1.7 months) and higher response rates (70.6% versus 11.1%) [38].

From a GUARDANT360^®^ database of NSCLC liquid biopsies, several *ALK* fusion partners could be identified: *EML4* (85.4%), *STRN* (6%), as well as *KCNQ, KLC1, KIF5B, PPM1B,* and *TGF*. Additionally, liquid biopsies from 31 patients had been analyzed upon tumor progression under ALK inhibition. Resistance alterations could be detected in 16 cases (53%), finding a number of 1–3 resistance mutations. Moreover, in 6 patients who had an initial *EGFR* activating mutation, the presence of an *ALK* rearrangement could be detected at progression [39].

In the Phase II/III blood first assay screening (BFAST) trial, 2.219 patients with advanced (stage IIIB or IV) NSCLC underwent FoundationOne^®^ Liquid CDx assay in order to identify actionable genomic alterations. *ALK* rearrangements were found in 119 patients (5.4%), and 87 out of 119 received treatment with alectinib. This subgroup showed an objective response rate of 87.4% (95% CI: 78.5–93.5%), with a response rate of 75.9% (95% CI: 63.6–88.2%) at 12 months and a median investigator-assessed PFS of 78.4% (95% CI: 69.1–87.7%) [40].

#### 2.2.5. MET

*MET* exon 14 skipping mutations and *MET* amplifications [41] predict response to targeted treatment with MET inhibitors, such as crizotinib [42], capmatinib [43,44], both FDA approved for NSCLC harboring *MET* exon 14 skipping mutations [45], or tepotinib [46,47]. Additionally, *MET* alterations can emerge as resistance mechanisms to other molecularly targeted agents [48].

As a consequence of the liquid biopsy testing in the FLAURA3 trial, distinct molecular aberrations could be uncovered as resistance events to treatment with osimertinib. These alterations included *MET* amplifications (15%), EGFR p.C797S mutation (7%), *HER2*-amplifications, as well as *PIK3CA* and *RAS* mutations (2–7%) [2].

DdPCR has been used to detect MET copy number alterations in cfDNA in a prospective study including 174 patients with several cancers, most frequently in metastatic stage. Concentration of plasma cfDNA correlated significantly with *MET* copy number. Additionally, *MET* amplifications were determined in CTCs, and longitudinal monitoring of both parameters, cfDNA and *MET* in CTCs, was performed during ongoing treatment with anti-EGFR therapy, as *MET* overexpression might also contribute to resistance to these drugs. As assessed by the Parsortix system, the presence of *MET*-positive CTCs (≥1) correlated with poorer overall survival in patients with head and neck cancers (HR = 6.66, *p* = 0.05). *MET* amplification assessment had a concordance of 91.67%, as compared with tissue biopsies evaluation by FISH (ratio MET/CEN−7 > 4). Plasma ddPCR had a sensitivity of 85.71%, as compared to tissue analysis, and a specificity of 100% [49].

In a comprehensive assessment of ctDNA from 1552 NSCLC patients, using hybrid capture-based genomic profiling, *MET* exon 14 skipping mutations were detected in 1.9% of cases. Moreover, additional activating SNVs of MET (p. L1195V, p. D1228H, and p. Y1230C) could be detected in three cases [50].

In a study analyzing the presence of *MET* alterations in ctDNA, assessed with NGS using GUARDANT360^®^, 438 patients with several solid tumors were screened. Additionally, in 263 patients matching tissue NGS, covering 182–315 genes, was performed. Here, 71.2% of patients had recurrent or metastatic disease, and the most frequent cancer types analyzed were gastrointestinal (28.1%), brain (24.9%), and lung tumors (23.2%). In addition, 31 patients (7.1%) showed alterations in *MET*, which correlated in a multivariant analysis with presence of bone metastases (*p*  =  0.007), as well as concomitant aberrations in *TP53* (*p*  =  0.001) and *PTEN* (*p*  =  0.003). Additionally, a shorter median time to metastasis or tumor recurrence was found to be associated with presence of *MET* alterations (1.0 vs. 10.4 months, *p*  =  0.044), as well as a shorter overall survival (30.6 vs. 58.4 months, *p* = 0.013). Tissue biopsies were available and screened for *MET* alterations in only 18 out of the 31 patients. Interestingly, these were found only in two tissue biopsies, so that the authors concluded that ctDNA was more sensitive for detection of *MET* alterations, and associated with poorer prognosis in the analyzed population [51].

#### 2.2.6. BRAF

The activating BRAF p. V600E mutation is present in approximately 1–2% of NSCLC patients [52]. The presence of the p. V600E mutation sensitizes to treatment with combinations of BRAF and MEK-TKIs [52,53]. In a case series of six patients with known BRAF p. V600E mutated NSCLC, cfDNA and CTCs were analyzed for the BRAF p. V600E mutation. Detection was performed using ddPCR, which identified the mutation in all six samples of cfDNA, however, only in one out of six samples extracted from CTCs [54]. In a study evaluating the performance of Competitive Allele-Specific TaqMan^®^ PCR (CastPCR) for detection of driver oncogenic mutations in plasma cfDNA from patients with lung adenocarcinoma, 107 patients were included, and plasma samples, as well as paired tissue biopsies, were collected. Using CastPCR, plasma samples were screened for the presence of *EGFR* and *BRAF* mutations. For the BRAF mutations (c.1406G>C p. G469A, c.1799T>A p. V600E, c.1781A>G p. D594G), the liquid biopsy test showed a sensitivity of 28.6% (2/7), a specificity of 93.0% (93/100), a concordance with the tissue detection of 88.8% (95/107), a PPV of 22.2% (2/9), and a NPV of 94.9% (93/98) [55].

In a case report, plasma from a patient with an *EGFR*-mutated advanced lung adenocarcinoma with initial response to osimertinib, followed by development of resistance and progressive disease, was analyzed with ddPCR. Liquid biopsy samples, as well as paired tissue samples, were interrogated for the presence of resistance mutations, such as EGFR p. T790M, EGFR p. C797S, BRAF p. V600E, *MET* amplification, and *HER2* amplification. The BRAF p. V600E mutation could be detected as resistance mechanism at time point of tumor progression under treatment with osimertinib. Additionally, the initial EGFR Del19 and p. T790M mutations could be further detected, illustrating the persistence of the initial subclone [56].

#### 2.2.7. ROS1

*ROS1* rearrangement can be found in approximately 1% of NSCLC patients [57], predicting response to TKIs, such as crizotinib [58], ceritinib [59], lorlatinib [60], entrectinib [61], or repotrectinib [62]. In a study including 128 patients with known *ALK* or *ROS1* alterations, liquid biopsy samples underwent genomic profiling using the InVisionFirst^®^-Lung, a ctDNA NGS platform assessing 37 genes. Of these patients, 101 had a known rearrangement in *ALK* and the other 27 in *ROS1*. For detection of fusions in *ALK* or *ROS1*, and at time point of diagnosis, the NGS assay showed a sensitivity of 67%. ALK rearrangement could be more frequently detected in patients progressing under TKI treatment, as compared to patients with response (46% vs. 11%). Resistance mutations to *ALK* inhibitors could be identified in 22% of patients, being ALK p. G1202R, the most frequently detected mutation. The ROS1 p. G2032R resistance mutation was found in 30% of evaluated cases. Patients with no detectable mutations in ctDNA had a longer median overall survival despite progression on TKIs [63].

#### 2.2.8. RET

RET rearrangements are found in 1–2% of patients with NSCLC [64], conferring potential therapeutic vulnerability to several TKIs, such as selpercatinib [65] or praseltinib [66]. In a comprehensive genomic analysis using GUARDANT360^®^, cfDNA of 32.989 patients with metastatic solid cancers was analyzed. Specifically, 176 *RET* alterations were found in 170 patients, corresponding 143 to fusions and 33 to missense mutations. Moreover, 125 patients out of 170 had NSCLC, followed by colorectal, breast, thyroid, and other cancers. *KIF5B-RET* fusions were found to be highly specific for NSCLC. The study also showed that patients with *RET* fusion partners distinct from *KIF5B*, exhibited also enrichment for alterations in the MAPK pathway and *EGFR* [67].

#### 2.2.9. KRAS

*KRAS* oncogenic mutations are present in 25–35% of NSCLC [68,69]. In a cohort of 194 patients, assessment of *KRAS* mutational status has been performed through a ctDNA plasma NGS analysis (SiRe^®^). *KRAS* mutations were detected in 18.6% of the screened cases, and for these patients, no further concomitant mutations have been found in *EGFR*, *NRAS*, *BRAF*, *KIT*, or *PDGFRA.* Moreover, 36.1% of the detected mutations corresponded to the currently targetable p. G12C KRAS mutation [70,71]. However, KRAS mutations can also coexist with further “targetable” oncogenic alterations [72,73,74]. Such presence of concomitant alterations to KRAS can be also used as a “positive control” of liquid biopsies. The only detection of *KRAS* in liquid biopsies can be of further use for longitudinal molecular monitoring of disease [75,76].

## 3. Case Series: Challenges of the Use of Liquid Biopsy in Daily Management of Lung Cancer Patients

### 3.1. Case 1

**Figure 1:**
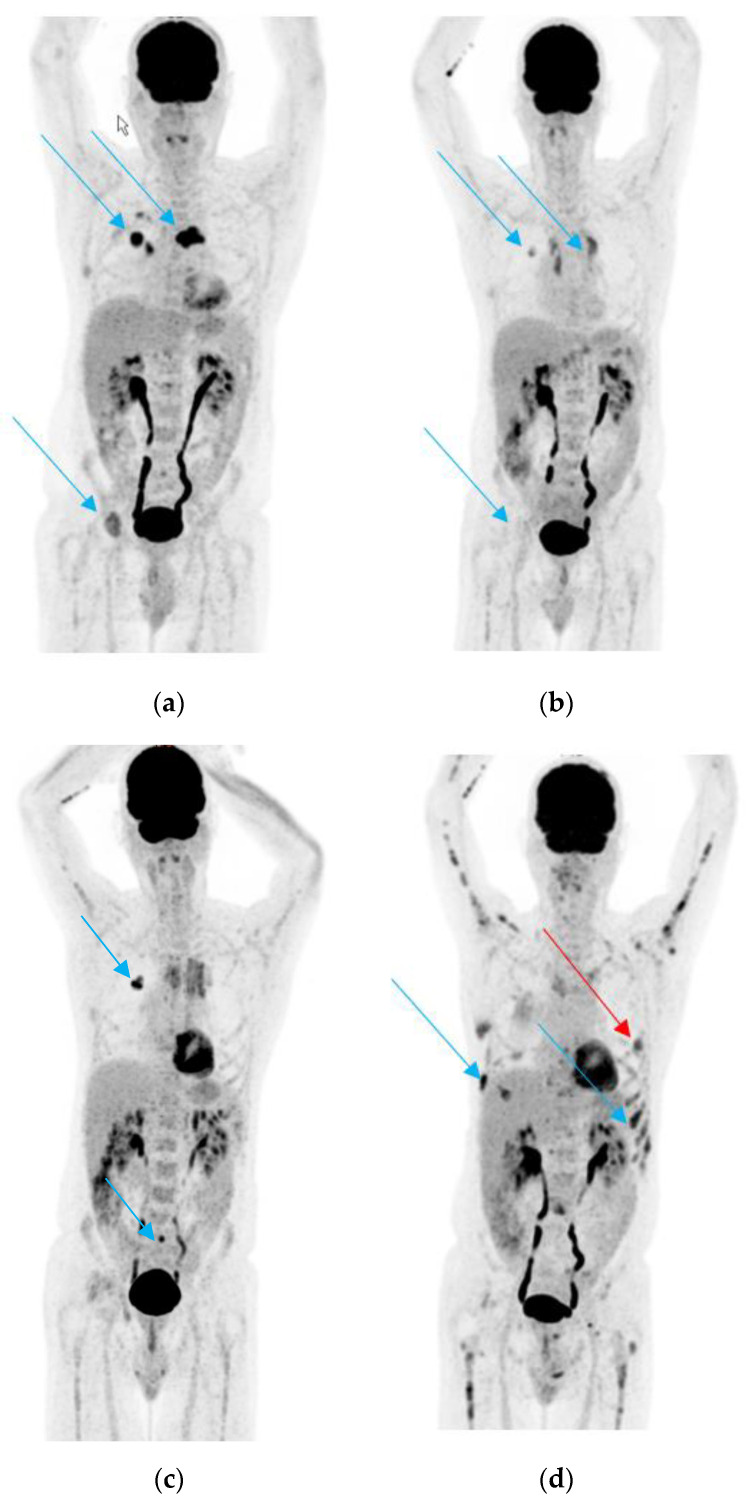
(**a**). ^18^F-fluorodeoxyglucose positron emission/computed tomography (^18^FDG-PET/CT) scan performed before start of treatment; the arrows show the primary tumor in the lung and some of the bone metastases. (**b**). ^18^FDG-PET/CT scan performed after 3 months of treatment with erlotinib and bevacizumab. The arrows show partial remission of the primary tumor and the bone metastases. (**c**). ^18^FDG-PET/CT scan performed after 9 months of treatment with erlotinib and bevacizumab. The blue arrows show the slight progression of the primary tumor and in the sacrum. (**d**). ^18^FDG-PET/CT in January 2019. The blue arrows show new diffuse bone metastasis of several skeletal regions and the red arrow a new pleural lesion, which was biopsied.

A 60-year-old male patient, never-smoker, was referred with the suspicion of metastatic NSCLC in June 2017. The initial staging with ^18^F-fluorodeoxyglucose positron emission/computed tomography (^18^FDG-PET/CT) showed a primary tumor in the right upper lobe and multiple bone metastases in the spine and right acetabulum (Figure 1a). The histological analysis of the primary tumor showed an adenocarcinoma expressing TTF-1, harboring the EGFR exon 21 p. L858R mutation.

The initial stage according to the TNM 8th Edition was cT1pN1cM1c (stage IVB). The patient started on treatment with erlotinib and bevacizumab, based on the results of the BELIEF study [77], as well as received radiotherapy to the symptomatic bone metastases in the spine and acetabulum. Additionally, antiresorptive treatment with monthly denosumab was initiated. The restaging ^18^FDG-PET/CT after 3 months of treatment showed a partial remission of the primary tumor and bone metastases (Figure 1b). Nevertheless, 9 months from treatment initiation, a slight progression of the primary tumor and appearance of new bone metastases in the sacrum were detected (Figure 1c). A rebiopsy of the primary tumor with targeted *EGFR* PCR using cobas^®^ EGFR Mutation Test v2 was performed, which showed the previously detected p. L858R mutation in exon 21 of *EGFR* and no other alterations.

The patient continued treatment with erlotinib and bevacizumab, but stereotactic radiotherapy to the oligoprogressing primary tumor and sacrum was initiated. The patient continued to be in partial remission until January 2019, when he presented with progressively increasing thoracic pain. The restaging ^18^FDG-PET/CT showed new diffuse lytic bone lesions in several skeletal regions and a new pleural lesion, with stable primary tumor (Figure 1d).

A transthoracic biopsy of the new pleural metastasis, as well as a plasma liquid biopsy using the Oncomine^TM^ Lung cfDNA Assay was performed. Liquid biopsy showed no *EGFR* mutations but the presence of the pathogenic BRAF p. V600E mutation. The biopsy of the pleural metastasis revealed an infiltrate of lambda-restricted plasma cells with aberrant CD117, CD56, and CyclinD1 coexpression, consistent with a plasmacytoma. Furthermore, the presence of the BRAF p. V600E mutation in the pleural metastasis was confirmed, as well.

**Figure 2:**
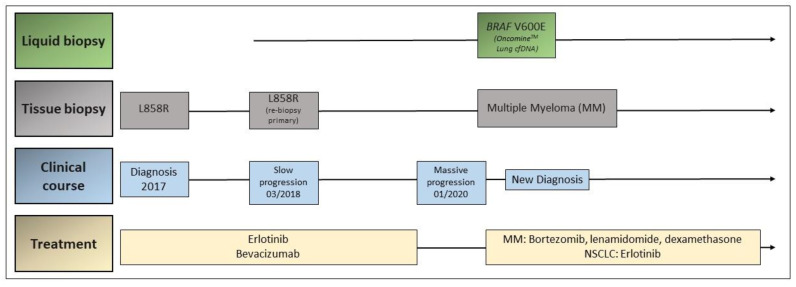
Timeline of disease and treatment course in case 1.

After further diagnostic work-up, including bone marrow examination, diagnosis of plasma cell myeloma (syn. multiple myeloma) IgG lambda type was made, and the patient started treatment with bortezomib, lenalidomide, and dexamethasone. The treatment with erlotinib for the metastatic NSCLC was continued, with discontinuation of bevacizumab. Currently, the patient is still on partial remission for both diseases (Figure 2).

### 3.2. Case 2

A 74-year-old female patient was diagnosed with lung adenocarcinoma with bilateral pulmonary disease (cT2a N3 M1a, stage IVA according to the TNM 8th Edition) in December 2015. The molecular analysis on tissue biopsy revealed the coexistence of a p. L858R mutation in exon 21 and an ab initio p. T790M mutation in exon 20 of the *EGFR* gene. The patient started on treatment with osimertinib in February 2016 achieving a partial response as best response by CT scan, with no significant toxicities.

In September 2017, asymptomatic bilateral pulmonary progression was detected. Liquid biopsy was negative for EGFR p. T790M mutation as assessed by cobas^®^ EGFR Mutation Test v2. In November 2017, an atypical segmentectomy of the lower right lobe was performed. The biopsy showed a mixed tumor with lepidic adenocarcinoma areas and small-cell lung cancer (SCLC). No *EGFR* mutations were detected in the molecular analysis of the tumor specimen. Osimertinib was then discontinued and the patient began treatment with carboplatin plus etoposide, with partial response.

In March 2018, a new asymptomatic pulmonary progression was detected by CT scan. Liquid biopsy with cobas^®^ EGFR Mutation Test v2 was repeated showing the presence of an EGFR p. T790M mutation. The patient refused treatment with osimertinib at that point. In August 2018, respiratory symptoms (dyspnea and cough) were progressing, so that treatment with erlotinib and bevacizumab was started.

**Figure 3:**
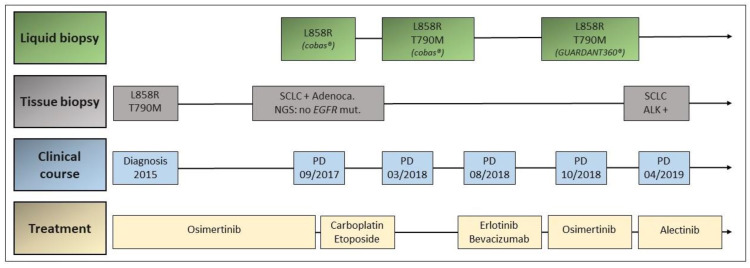
Timeline of disease and treatment course in case 2. Adenoca.: adenocarcinoma; mut.: mutation; NGS: next-generation sequencing; PD: progressive disease; SCLC: small-cell lung cancer.

A further pulmonary progression was detected by CT scan in October 2018. A plasma test with cobas^®^ EGFR Mutation Test v2 was performed, which showed the coexistence of p. L858R and p. T790M EGFR mutations. With this result, treatment with osimertinib was reinitiated, with partial response and no major toxicities. In April 2019, a new pulmonary and hepatic progression was detected by CT scan. A hepatic biopsy was performed with the result of high-grade neuroendocrine carcinoma with an ALK rearrangement detected by VENTANA ALK (D5F3) CDx Assay^®^. The coexistence of p. L858R and p. T790M EGFR mutations was still detected by cobas^®^ EGFR Mutation Test. Treatment with alectinib was started in April 2019. One week after treatment initiation, the patient died due to respiratory insufficiency and a syndrome of inappropriate antidiuretic hormone secretion (SIADH) (Figure 3).

### 3.3. Case 3

**Figure 4:**
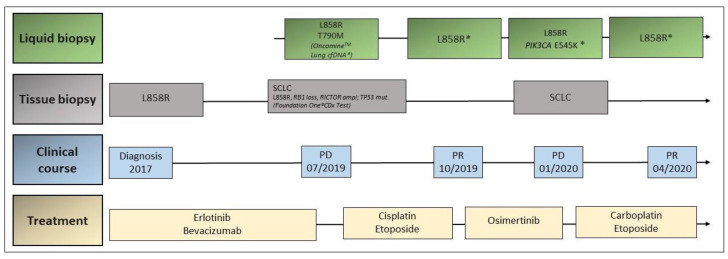
Timeline of disease and treatment course in case 3. Ampl.: amplification; mut.: mutation; PD: progressive disease; PR: partial response; SCLC: small-cell lung cancer.

A 65-year-old female patient was diagnosed with pleural metastatic lung adenocarcinoma in November 2017. A p. L858R mutation in exon 21 of *EGFR* was detected in the pleural biopsy, so that the patient started on treatment with erlotinib and bevacizumab in December 2017. Following morphologic and metabolic response, the tumor progressed in the pleura about 20 months after treatment initiation. A liquid biopsy with the Oncomine^TM^ Lung cfDNA Assay was performed, which revealed the presence of the p. T790M EGFR resistance mutation, as well as the original p. L858R mutation. Additionally, a pleural biopsy was performed, showing histologic transformation into SCLC. Hybrid NGS using FoundationOne^®^ CDx Test was performed on this biopsy revealing the presence of the original EGFR p. L858R mutation, as well as *RB1 loss*, *TP53* mutation, and *RICTOR* amplification, among others. The patient started on cisplatin and etoposide in August 2019, achieving a partial response in ^18^FDG-PET/CT scan performed in October 2019. At this point, liquid biopsy by Oncomine^TM^ Lung cfDNA Assay showed the presence of the EGFR p. L858R mutation, but p. T790M and *TP53* mutations could not be detected. The patient refused to receive further chemotherapy and a treatment with osimertinib was started due to the presence of the EGFR p. T790M mutation at the first tumor progression. The ^18^FDG-PET/CT scan performed 2 months after starting osimertinib and 3 months after initial response showed tumor progression at different locations (pleura, lung, and lymph nodes). Plasma test with Oncomine^TM^ Lung cfDNA Assay found the persistent EGFR p. L858R mutation, as well as a new PIK3CA p. E545K mutation. A biopsy of the parasternal lymph node metastasis showed SCLC. Chemotherapy with carboplatin and etoposide was, therefore, restarted in February 2020, and a new liquid biopsy by Oncomine^TM^ Lung cfDNA Assay reported the reappearance of the known EGFR p. L858R mutation, without any additional findings. Last tumor assessment revealed a mixed response with oligoprogression of lymph node metastases and new bone metastases. The patient is currently undergoing local treatment with radiotherapy (Figure 4).

## 4. Discussion

Unlike tissue-based tumor molecular analysis, liquid biopsy allows minimally invasive genomic profiling and, if performed serially, may provide a more comprehensive information regarding global tumor evolution occurring at different metastatic sites throughout the course of disease. Additionally, liquid biopsy can be a useful tool for detection of emerging resistant tumor subclones [64,78,79].

However, the here reported clinical cases also illustrate the current limitations of liquid biopsy as compared to the tumor rebiopsy. In case 1, in the absence of tumor tissue biopsy, the presence of a *BRAF* mutation in cfDNA might have been interpreted as an underlying resistance mechanism to the EGFR inhibition [80,81]. This solely information might have compromised the diagnosis and treatment of multiple myeloma. However, the data proceeding from tissue and liquid biopsy can still be complementary, especially for progressive and refractory diseases, where increasing molecular tumor heterogeneity derived from coexisting distinct molecular subclones occurs.

This also underlines that the heterogeneity of assays might lead to major challenge, especially in regard to the spectrum of covered mutations and analytical sensitivity of the different liquid biopsy platforms. For this reason, the selection of the most optimal assay, as well as a comprehensive interpretation of results, is crucial. To overcome such challenge, the access to a molecular tumor board for multidisciplinary assessment of individual clinical situations and interpretation of obtained data could be fundamental.

Since multiple liquid biopsy techniques are available (RT-PCR, ddPCR, and NGS), we give here an overview of their main advantages and limitations (Table 1). The most frequently used assays for common genomic alterations in NSCLC are summarized in Table 2.

A clear example of such variety of tests, is the development of multiple liquid biopsy techniques and platforms to assess activating and resistance mutations in the *EGFR* gene. The complexity of data interpretation derives not only from the broad spectrum of tests, but also from the different sensitivity, specificity, and concordance with tissue-results of each assay. Even if the same platform is used, variations of mutations can be detected when different patients’ populations are evaluated [82]. In a cohort of 102 patients with *EGFR* mutant lung cancer, the real-time PCR by cobas^®^ EGFR Mutation Test v2 has shown a concordance for EGFR p. L858R and exon 19 deletion to matched tissue samples of 87.4%, a sensitivity for detection of the *EGFR* mutations of 70.6%, and a specificity of 91.7%. Two additional cohorts (n = 238 from the FASTACT-2 study and n = 196 published by Weber et al.), using cobas^®^ EGFR Mutation Test v2, reported a specificity of 96%, sensitivity of 60–75%, and a concordance with tissue biopsy of 88–91% [83,84,85]. NGS-based liquid biopsy assays, such as Oncomine^TM^ Lung cfDNA Assay or GUARDANT360^®^ Circulating Tumor DNA Assay, have shown sensitivities for detection of covered genomic alterations ranging from 58% to 87%, with specificities up to 99–100% [86,87].

Moreover, a further limitation of the liquid biopsy is related to the possibility of sampling bias and limited sensitivity of some assays. For example, the sensitivity for detection of the resistance p. T790M EGFR mutation in liquid biopsies is lower when compared to the initial activating *EGFR* mutations [1], and a tissue rebiopsy is still recommended when p. T790M is not detected in plasma, especially if the initial activating *EGFR* mutation is also absent [82].

Despite the lower sensitivity, a potential advantage of NGS-based liquid biopsy assays could be the simultaneous detection of other coexisting mutations, also when their presence has no direct therapeutic consequence. This is the case of concurrent *KRAS* mutations [72,73,74], which, with the exception of KRAS G12C [70,71], are currently not targetable. Their detection can, however, serve as a positive sampling control and for disease course monitoring.

The cases 2 and 3 illustrate the importance of optimal assay selection for disease monitoring in patients undergoing targeted treatment. In contrast to RT-PCR or ddPCR platforms, NGS-based assays allow simultaneous screen and discovery of multiple possible resistance mechanisms, giving more information about course of disease and, in our opinion, should be prioritized, whenever possible. In this way, uncovered emerging resistance alterations may offer novel therapeutic opportunities for personalized molecularly targeted treatment. In case 2, liquid biopsies during treatment with osimertinib had been performed with cobas^®^ EGFR Mutation Test v2, a RT-PCR assay, whereas liquid biopsy NGS with Oncomine^TM^ Lung cfDNA Assay was used in case 3. In case 2, cobas^®^ EGFR Mutation Test v2 would have missed the emergence of the p.C797S mutation, one of the main resistance mechanisms reported under treatment with third-generation EGFR TKIs, especially osimertinib [88]. However, some RT-PCR-based assays, such as EntroGen ctEGFR Mutation Detection Kit, do cover this relevant resistance mutation. Though not yet drugable, the screening for the p.C797S mutation will have an increasing relevance in the years, since treatment for third-line TKIs has moved to first-line treatment for *EGFR*-mutated NSCLC [36].

Our third case illustrates the potential of liquid biopsy to be implemented as a noninvasive tool for monitoring disease course and response or development of resistance to treatment, complementary to imaging. In a very exhaustive manner, Rolfo et al. reviewed multiple available data regarding the monitoring of the p. T790M EGFR mutation with different assays [82]. Its detection in plasma is considered sufficient to switch the TKI treatment.

However, as for the second and third cases, liquid biopsy missed SCLC transformation and, therefore, tissue biopsy is crucial when the liquid biopsy is not informative or histologic transformation is suspected based on clinical or radiological discordance, in line with previously reported cases [89,90,91]. Interestingly, Minari et al. reported that a detected low ratio p. T790M/*EGFR* activating mutation in liquid biopsy correlated with the presence of histological transformation to SCLC [91], which might be useful whenever a tissue biopsy is not feasible.

In our review, we analyze the main pros and limitations of the distinct liquid biopsy assays, underscoring the difficulty and the relevance of the optimal assay selection. Additionally, we present the first reported case of a concomitant multiple myeloma diagnosis, uncovered through liquid biopsy in a patient with NSCLC.

In summary, liquid biopsy is a rapidly developing minimally invasive diagnostic tool with great potential to detect oncogenic drivers at baseline and along tumor evolution. However, this technique has limitations that should be taken into account in the clinical practice. The complexity of results interpretation entails the need of multidisciplinary approach.

## 5. Conclusions

Liquid biopsy has become an essential part of diagnostic genomic profiling and dynamic monitoring of disease course in lung cancer, enabling a better characterization of the molecular heterogeneity and clonal evolution that emerges during tumor progression and as adaptive response to targeted oncological treatment. Nevertheless, tissue biopsies still remain essential and provide *gold standard* information regarding tumor molecular characterization and are especially necessary to detect histologic transformation, which can occur in the setting of resistance to targeted therapy.

## Figures and Tables

**Table 1:** Liquid biopsy assays with main advantages and limitations. Abbreviations: ddPCR: digital droplet PCR.

Assay Type	Advantages	Main Limitations
**Real-time PCR**	-Rapid results -Cost effectiveness	-Limited number of DNA sequences -Allele frequency of at least 1% for some assays (Cobas^®^ EGFR Mutation Test v2, Therascreen)
**Digital droplet PCR**	-High sensitivity (detection of low allele frequency, 0.005–0.1%)	-Limited number of DNA sequences
**Next-generation sequencing**	-Larger number of DNA sequences -Detection of coexisting mutations	-Higher allele frequency required compared to ddPCR (0.1%) -Higher costs -More complex data analysis and interpretation

**Table 2:** Main target genes and mutations in non-small cell lung cancer (NSCLC) and summary of most frequently used liquid biopsy assays used for their assessment. Abbreviations: ddPCR: digital droplet PCR; NGS: next-generation sequencing; RT-PCR: real-time PCR; * e.g., Oncomine™ Lung cfDNA Assay, FoundationOne^®^ Liquid CDx, GUARDANT360^®^, InVisionFirst^®^-Lung).

Target Genomic Alteration		Most Frequent Liquid Biopsy Assays		Relevant Features
*EGFR*	Del19 and p. L858R	RT-PCR	-Cobas^®^ EGFR Mutation Test v2 -Idylla^TM^ ctEGFR Mutation Test -EntroGen ctEGFR Mutation Detection Kit	-Cobas^®^: 79% concordance with positive tissue test (FLAURA trial)
	p. T790M	RT-PCR	-Cobas^®^ EGFR Mutation Test v2 -Idylla^TM^ ctEGFR Mutation Test -EntroGen ctEGFR Mutation Detection Kit	-Cobas^®^: 51% concordance with positive tissue test (AURA3 trial) -Relevance of tissue rebiopsies in case of negative plasma test
		Droplet Digital™ PCR		-71% vs. 41% sensitivity, 83% vs. 100% specificity as compared to Cobas^®^, and 74% concordance with tissue test (AURA trial, 38 samples)
		BEAMing digital PCR		-81% vs. 73% sensitivity, 58% vs. 67% specificity as compared to Cobas^®^ (AURA trial, 72 samples)
		NGS *		-For GUARDANT360^®^ 70-gene NGS: 85% concordance with positive tissue test (Therascreen EGFR RGQ PCR Kit, Qiagen)
	p. C797S	RT-PCR (EntroGen ctEGFR Mutation Detection Kit)		
		ddPCR or NGS*		
*ALK*	Fusions and mutations	RT-PCR		-From plasma or platelets (65–79% sensitivity, higher for platelets; 89–100% specificity)
		NGS *		-Allows identification of distinct fusion partners
*MET*	Exon 14 skipping and amplifications	ddPCR or NGS *		-ddPCR for MET amplification: 86% sensitivity and 100% specificity as compared with positive tissue FISH
*BRAF*	Mutations	RT-PCR	-Competitive Allele-Specific TaqMan^®^ PCR	-29% sensitivity, 93% specificity, and 89% concordance as compared with positive tissue test
			-Idylla^TM^ ctBRAF Mutation Test	
		ddPCR or NGS *		
*ROS1*	Fusions and mutations	NGS *		-InVisionFirst^®-^Lung: 67% sensitivity for ROS1/ALK fusions at diagnosis
*RET*	Fusions and mutations	NGS *		-Allows identification of distinct fusion partners
*KRAS*	Mutations	RT-PCR, ddPCR, or NGS *		
